# Effects of Ankle Stabilization Exercises Using Sonic Balance Pad on Proprioception and Balance in Subjects with Ankle Instability

**DOI:** 10.3390/healthcare11182544

**Published:** 2023-09-14

**Authors:** Merve Nur Uygun, Dong-Kyu Yang, Jung-Su Moon, Dae-Sung Park

**Affiliations:** Department of Physical Therapy, Konyang University, Daejeon 35365, Republic of Korea; 22804638@konyang.ac.kr (M.N.U.); 18811028@konyang.ac.kr (D.-K.Y.); 18811018@konyang.ac.kr (J.-S.M.)

**Keywords:** ankle instability, balance training, proprioception

## Abstract

Sound waves generate acoustic resonance energy that penetrates deeply and safely into body areas normal mechanical vibrations cannot reach. The sonic balance pad utilizes these sound waves to create an optimal musculoskeletal response. The purpose of this study was to investigate the effects of a 4-week ankle stabilization exercise program using a sonic balance pad on proprioceptive sense and balance ability in individuals with ankle instability. This study was conducted as a randomized control-group pre-and post-test design in 30 participants (21 females and 9 males) who had experienced an ankle fracture or sprain within the last 5 years or who scored 11 points or more on The Identification of Functional Ankle Instability. The ankle stabilization exercise program was conducted for 4 weeks in the experimental group (*n* = 15), to which sonic pads were applied, and the control group (*n* = 15), to which balance pads were applied. All participants were assessed for their intrinsic proprioceptive sense of dorsiflexion and plantarflexion, static balance test, dynamic balance test, and long jump test were measured before and after 4 weeks as dependent variables. After 4 weeks of training, a significant difference was shown in the right dorsiflexion error (Balance pad = PRE: 2.47 ± 0.92; POST: 2.33 ± 1.40, Sonic pad = PRE: 3.27 ± 1.39; POST: 1.20 ± 0.77) and the left plantar flexion error (Balance pad = PRE: 2.00 ± 1.36; POST: 2.73 ± 1.22, Sonic pad = PRE: 3.53 ± 1.25; POST: 2.20 ± 1.01) (*p* < 0.05) between the experimental and control groups in the proprioception test. In the static balance test, there was no significant difference between the experimental and control groups during the pre, post, and variation stages. However, in the Y-Balance test, which is one of the dynamic balance tests, there was a significant difference between the experimental and control groups at various points, including anterior left (Balance pad = PRE: 72.85 ± 19.95; POST: 63.41 ± 8.66, Sonic pad = PRE: 68.16 ± 6.38; POST: 76.17 ± 3.67), posteromedial right (Balance pad = PRE: 78.59 ± 15.34; POST: 81.41 ± 10.37, Sonic pad = PRE: 86.33 ± 16.44; POST: 102.23 ± 11.53), posteromedial left (Balance pad = PRE: 78.00 ± 16.99; POST: 83.36 ± 10.15, Sonic pad = PRE: 88.96 ± 19.92; POST: 102.45 ± 12.98), posterolateral right (Balance pad = PRE: 78.16 ± 14.33; POST: 82.61 ± 10.73, Sonic pad = PRE: 87.95 ± 17.51; POST: 101.34 ± 15.37), and posterolateral left (Balance pad = PRE: 80.86 ± 14.96; POST: 81.31 ± 7.16, Sonic pad = PRE: 91.23 ± 17.35; POST: 104.18 ± 11.78) (*p* < 0.05). Moreover, in the single-leg long jump test, which is another dynamic balance test, the experimental group (Sonic pad = PRE: 100.27 ± 29.00; POST: 116.80 ± 28.86) also demonstrated a significant difference in the right single-leg long jump compared to the control group (Balance pad = PRE: 91.87 ± 17.74; POST: 97.67 ± 17.70) (*p* < 0.05). When a sonic balance pad using sound waves was applied in addition to a 4-week ankle stabilization exercise program for participants with ankle stability, it helped to improve proprioception and dynamic balance ability.

## 1. Introduction

Ankle inversion sprains are the most prevalent musculoskeletal injury in sports and activities of daily living that most concern young physically active individuals [1]. It has been estimated that the incidence is about one ankle inversion per 10,000 people per day in the United States [2]. It is known that at least 73% of individuals who suffer an ankle sprain develop residual symptoms that increase the chances of re-injury and development of chronic ankle instability (CAI) [3]. CAI commonly consists of reoccurring ankle sprain, residual symptoms such as pain, giving way, and compromised proprioception and neuromuscular control, and encompasses functional and mechanical ankle instability [3,4]. Mechanical instability refers to objective measures of ligament laxity. Functional instability is defined as repetitive sprains and a feeling of giving way. Associated causal factors include proprioceptive deficit, muscle weakness, and lack of coordination [5,6]. Conservative treatments such as rehabilitation, taping, and bracing for the treatment of CAI can effectively reduce the recurrence of ankle sprains. On the other hand, strengthening the muscles around the ankle with well-planned proprioceptive exercises helps patients return to normal life and sport activities. Surgical treatment should only be used when conservative treatment has failed [7,8].

Balance training is used as one of the most effective interventions in various stages of CAI rehabilitation and is usually performed on an unstable support surface such as both sides utilized (BOSU) balance trainer or Aerobic step (STEP). This training increases muscle co-contraction, stiffness, joint stability, and balance, and improves mechanoreceptors. Exercise programs conducted on unstable surfaces have the potential to elicit adaptive responses that enhance postural control, balance, and functional ability within the neuromuscular system of individuals with ankle instability. This underscores that, beyond conventional exercise training, training on unstable surfaces yields a more positive impact on individuals [9,10].

Quiet stance under different foot positions has been studied to identify balance performance. Standing on an unstable surface provides a convenient method to increase the difficulty of the balance task and allows discrimination between healthy individuals and individuals with balance disorders. Standing on an unstable surface changes body orientation and foot pressure distribution and affects joint receptors and cutaneous mechanoreceptors in the foot. At the same time, using different pad types increases the difficulty of maintaining balance [11,12]. Training on an unstable surface alone is not sufficient to stimulate the ankle proprioceptors. Vibration stimulation combined with an unstable surface will be more effective in ankle rehabilitation [13].

The effect of vibratory stimulation on the neuromuscular system has been studied in different therapeutic and rehabilitative fields and has evolved into full-body training known as Whole Body Vibration (WBV) [14]. WBV training is neuromuscular training performed on an oscillating vibration platform that activates muscle spindles to facilitate the tonic vibration reflex. This training also improves α motor neuron excitability and synchronization of motor units to increase motor control in the ankle [15]. People with CAI have neuromuscular and proprioceptive deficits. Due to the strong sensory stimulus and activation of alpha-motor neurons, WBV training can also improve strength proprioception and balance in ankle instability [9,15].

Sound waves have many physiological benefits to the human body [16]. Acoustic vibration is a vertical vibration method using a sonic activator and refers to a sound wave transmitted by the vibration of air or characteristic material. It has the advantage of vibratory stimulation from a low frequency of 1 Hz to a high frequency of 100 Hz and can transmit vertical vibration toward the human body. This type of vibration stimulation induces blood flow and hormonal response, which has a positive impact on health [16,17]. Acoustic vibration provides the best relief of pain in the 60–600 Hz frequency band and is known as a method that prevents the deterioration of physical strength and muscle strength, applied to patients who do not exercise for a long time after surgery [18]. Although there are very few instruments that apply sound waves to balance pads, if the beneficial sound waves of the human body and balance pads are applied simultaneously, the effect of balance exercise will be different from existing balance instruments [16,18,19].

It is known that exercise programs accompanied by vibration stimulation are effective in rehabilitation treatment and improve balance ability and proprioceptive sense [9,19,20]. However, there appears to be a lack of previous studies on whether rehabilitation therapy using sonic vibration is effective in improving balance ability. The purpose of this study was to investigate the effects of a 4-week ankle stabilization exercise program using a sonic balance pad on proprioceptive sense and balance ability in individuals with ankle instability.

## 2. Methods

### 2.1. General Characteristics

A total of 30 subjects (9 males and 21 females) participated in this study, including a balance pad groups (*n* = 15, aged 20.33 ± 1.18 years, weight 57.93 ± 7.65 kg, height 162.8 ± 5.98 m, 2 males and 13 females) and a sonic balance pad group (*n* = 15, aged 21.27 ± 1.47 years, weight 62.13 ± 13.23 kg, height 164.73 ± 10.91 m, 7 males and 8 females). A recruitment notice was posted on K University’s notice board outlining the study content, location, inclusion and exclusion criteria, as well as potential risks and benefits, and the announcement also stated that 30 people who meet the defined conditions were to be recruited. The subjects were selected using the convenience sampling method. The inclusion criteria for participation in this study were adults 19 years of age and older, a history of ankle sprains in the past 5 years, and a score of 11 points or higher on the Identification of Functional Ankle Instability (idFAI) questionnaire [21]. The exclusion criteria were participation in a regular exercise program at least three times a week, severe pain in load-bearing joints even with mild exercise, and cardiopulmonary anomalies. We also excluded participants who may have been recently exposed to whole-body or other vibration machines just before or during the study period, and participants who were already receiving physical therapy. The purpose and procedure of the study were explained to all participants, and participants who did not provide written informed consent were also excluded from the study. This study was approved by the K University Institutional Review Board (IRB) (KYU 2021-09-017).

### 2.2. Procedures

**Figure 1:**
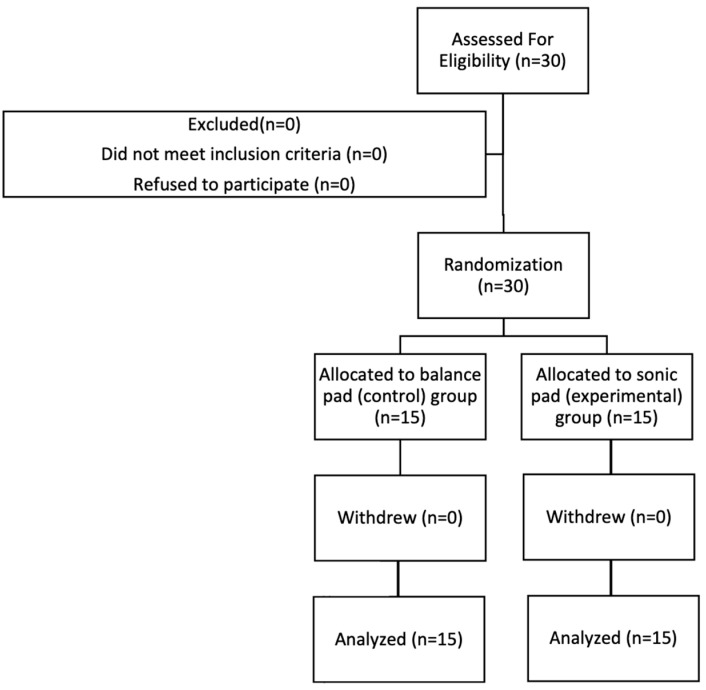
Flow of participants through the intervention.

This study was conducted as a pre-post randomized controlled trial design. Thirty subjects took part in this experiment. These subjects were assigned to one of two groups by concealed random allocation, using random numbers generated by online software (www.randomized.com): the control group (*n* = 15), and the experimental group (*n* = 15) (Figure 1). The researchers who applied and monitored the training protocol were different people. A complex exercise program consisting of proprioceptive exercise and muscle strengthening exercise was applied to both the experimental group and the control group to improve balance ability. All exercises were performed on the unstable surface and were the same for both experimental groups. Participants in the control group trained with a balance pad (AIREX^®^ Balance-pad, Switzerland), whereas participants in the experimental group trained with a sonic balance pad (EVOSONIC, Wonju, Republic of Korea). The exercise program was applied three times a week, approximately 30 min/time, for a total of 4 weeks. Proprioceptive function, static postural balance, and dynamic postural balance were tested in all participants before and after the exercise program.

## 3. Training Protocol

### 3.1. Ankle Stabilization Exercise Program

Warm-up exercises were performed on the subjects to prevent exercise-related injuries. After a 5 min standardized warm-up, three sets of 1 min brisk walking were performed. A 10 s rest was given between each set to prevent fatigue [22,23].

### 3.2. Proprioceptive Strengthening Exercise

**Figure 2:**
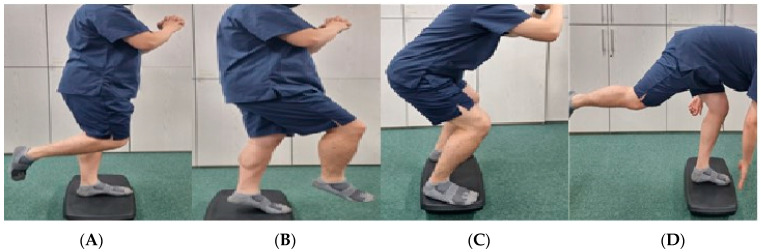
Ankle stability exercise ((**A**) one leg standing, (**B**) one leg heel-up, (**C**) squat, (**D**) one leg dead lift).

The exercise time for both groups on an unstable surface (balance and sonic pad) is approximately 15 min. For the first exercise, subjects stood on a balance pad (control group) or sonic balance pad (experimental group) and maintained their balance while keeping the knee joint of one leg bent 90 degrees without support. During the exercise, they clasped both hands at chest level to maintain the correct posture [24,25] (Figure 2A). In the second exercise, unlike the first exercise, they stood on one leg, slightly bending the hip joint and knee joint. While in this position, they raised the heel of the supporting leg and lowered it again [24,25,26] (Figure 2B).

### 3.3. Strength Training

For the third exercise, subjects stood on a stability pad or a sonic stability pad and had both feet shoulder-width apart as they felt comfortable. During the exercise, they clasped both hands at chest level to maintain the correct posture. Then, they moved downwards, pulling their hips back as if they were sitting in a chair. They moved down until their thighs were parallel to the floor and then up again [15,24,25,26,27] (Figure 2C). For the fourth exercise, the subject stood on a stability pad or sonic stability pad and lifted the non-weight-bearing leg to make a 90-degree bend at the hip joint and knee joint. In this position, they extended their legs back and at the same time bent their upper body forward until their legs and upper body were in a straight line. As their upper body gradually descended, they returned to their starting position without their hands touching the ground as their body became a “T-shaped” pattern [24,27,28,29] (Figure 2D). The cool-down exercise was performed for 3 min to remove fatigue substances accumulated during exercise [22,23]. The subjects self-massaged their muscle mass using a foam roller and a massage ball. In addition, calf muscle stretching was performed only for those who wanted additional stretching (Table 1).

## 4. Assessment

### 4.1. Proprioceptive Sensory Function

**Figure 3:**
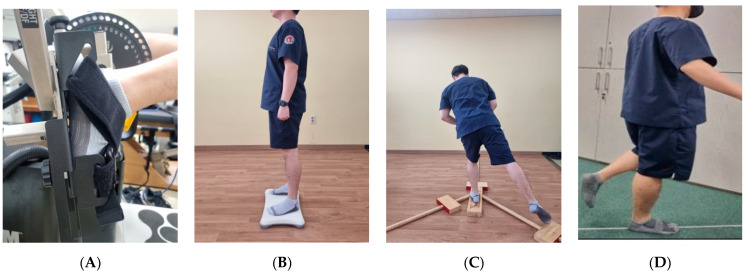
Assessment ((**A**) Proprioception, (**B**) Static standing balance, (**C**) Dynamic standing balance, (**D**) Single leg long jump).

The proprioceptive sensations of dorsiflexion and plantar flexion of the ankle were tested using a Cybex isokinetic dynamometer (Cybex NORM^®^, HUMAC, CA, USA). Gravity compensation of the Cybex isokinetic dynamometer was activated to achieve standardization before testing. Measurements made without gravity compensation of the device were considered invalid. Subjects performed the test twice with their eyes closed, after setting the angular velocity to 5°/s while in the supine and prone positions on the instrument. They held their feet at the set target angle for 10 s and returned to their original position. After performing the movement, the subjects were asked to find the same position themselves. The difference between the target angle and the angle performed by the subject was calculated and the average of the two repeated values was recorded on the right and the left. This measurement tool has high reliability (Intraclass correlation coefficient; ICC = 0.76–0.82) (Figure 3A) [30].

### 4.2. Static Balance Ability

A Wii Balance Board (Nintendo, Kyoto, Japan) was used to test static balance ability. After standing with both feet on the Wii Balance Board, the subject maintained an X-shaped posture by holding the opposite shoulder with both arms. In order to control the occurrence of postural fluctuations due to gaze, the subjects were asked to look at the picture in front at eye level while the eyes were open. Static balance was measured for 30 s after the subject took a stable stance on the Wii Balance Board. The subject took the same posture as before with eyes closed and measurement was taken again for 30 s. If the subjects lost their balance and fell off the Wii balance board during the measurement, the measurement was considered invalid. The measured values were analyzed using balance software (Balancia ver. 2.5, Mintosys, Seoul, Republic of Korea). The analyzed results measured the distance and velocity of movement of the center of pressure (COP) in the X and Y axes. The intra-rater reliability of the Balancia program is as high as ICC = 0.79–0.93, and the inter-rater reliability of the Balancia program is as high as ICC = 0.79–0.96 (Figure 3B) [12,31].

### 4.3. Dynamic Balance Ability

The Y-balance test (YBT) and single leg long jump test were used to test dynamic balance ability. The YBT is an abbreviated version of the star excursion balance test and is used to test the anterior, posteromedial, and posterolateral reach. Leg length was measured from the lower part of the anterior superior iliac spine to the medial malleolus bone. A Functional Movement Screen test kit was used to measure the distance the subject reached. The value obtained by dividing the average of three repeated measurements of each leg in all directions by the leg length was recorded as a percentage. The test was considered invalid when the subjects lost their balance while extending their extremities in the intended reaching direction and could not perform the targeted movement. This measurement tool used for the YBT has high reliability (ICC = 0.99) (Figure 3C) [32,33]. The single-leg long jump test is one of the methods used to test dynamic balance ability as well as agility. The single-leg long jump is a test in which the athlete jumps a long distance forward while maintaining a standing position on one foot. The test was considered invalid if the subjects could not maintain their position and lost their balance while landing after performing the single-leg long jump. The subjects were prepared by aligning the tips of their toes to the starting line. The subjects jumped forward while on one foot from their position, and the point where the subject’s heel touched the ground was recorded as the measurement value. The average of the values measured twice for each leg was recorded (Figure 3D).

## 5. Statistical Analysis

Data were analyzed using SPSS (Version 18.0, SPSS Inc., Chicago, IL, USA) and significance level was set at *p* < 0.05. Values are expressed as mean ± SD. All the measures were normally distributed, as determined by the Shapiro–Wilk test. The paired-sample *t*-test was used to compare pre- and post-intervention within the group. The independent sample *t*-test was used to compare the change between groups pre- and post-intervention.

## 6. Results

### 6.1. Participants

The participants of this study were 30 students (21 females and 9 males) at K University in Daejeon Metropolitan City, who understand the purpose and content of the study and voluntarily participated in the experiment. The basic characteristics of the participants are shown in Table 2. There was no significant difference in the basic characteristics of participants (age, height, and weight) in the experimental and control groups.

### 6.2. Proprioception Test

The pre-and post-intervention values showed significant differences in all values except for left plantar flexion errors (LPE) in the balance pad group and right plantar flexion errors (RPE) in the sonic pad group (*p* < 0.05). The independent sample *t*-test results showed a statistically significant difference in the LPE and right dorsiflexion error (RDE) (*p* < 0.05) (Table 3).

### 6.3. Static Balance Test

The paired samples *t*-test was used to find the difference within groups pre- and post-intervention, and the independent samples *t*-test was used to find the difference in variation between groups, and the results did not show a significant difference (Table 4).

### 6.4. Dynamic Balance Tests

#### 6.4.1. Y-Balance Test

The paired-sample *t*-test showed that the balance test scores between pre-and post-intervention were statistically significant differences in all values except the anterior left (AL) and posterolateral left (PLL) items in the balance pad group (*p* < 0.05). The independent sample *t*-test results indicated a statistically significant difference in the three components of balance (anterior right (AR), posteromedial right (PMR), PLL directions) between the changes in the experimental group and the changes in the control group at *p* < 0.05 (Table 5).

#### 6.4.2. Single Leg Long Jump Test

**Figure 4:**
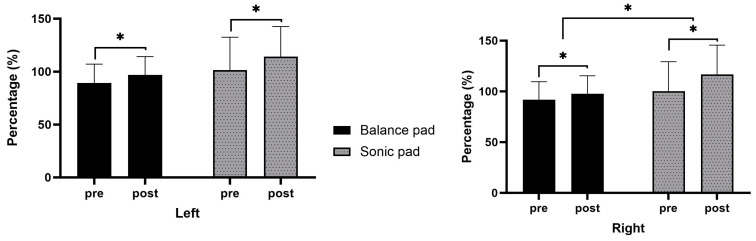
The comparisons of long jump (*n* = 30) (*: *p* < 0.05).

The results further revealed statistically significant differences between the pre- and post-intervention scores of the single leg long jump test (*p* < 0.05). Similarly, a statistically significant difference was found in the right single-leg long jump category as a result of an independent sample *t*-test between groups (*p* < 0.05) (Figure 4).

## 7. Discussion

The purpose of our project was to investigate the effect of an exercise program using sonic vibration on the improvement of proprioception and balance ability in patients with ankle instability. We applied a 4-week ankle stabilization exercise program to the experimental group using the sonic pad and the control group using the balance pad. The result of our study showed that the amount of change in the sonic balance pad group was significantly reduced compared to the balance pad group in all variables except LDE and RPE in the proprioceptive test. As a result of the YBT, there was a significant increase in the sonic balance pad group compared to the balance pad group in all variables except PML and PLR between the groups. As a result of the long jump test, the amount of change in the sonic balance pad group increased significantly compared to the balance pad group in all variables except the left long jump. This is thought to be the result of the concurrent application of sonic vibration to unstable support surface training.

Chronic ankle instability can lead to balance and walking problems due to weakness of the ankle muscles and decreased proprioceptive sense [34]. It has been proven by research that an exercise program is effective in improving the balance ability of individuals with chronic ankle instability. According to Linens et al. (2016) [35], 34 adults with ankle instability were trained to stand on one leg on a balance board for 4 weeks. As a result, the foot lift measure of static postural control significantly improved in the rehabilitation group (from (5.61 ± 2.51) to (3.82 ± 2.18)) compared with the control group (from (5.00 ± 1.68) to (4.61 ± 1.72)). The foot lift test, which evaluates static balance, bears similarities to the one-leg deadlift exercise performed in this study. Therefore, it can be inferred that this exercise likely yields a similar effect on static balance.

Cruz-Diaz et al. (2015) [32] demonstrated improvements in ankle instability according to the Cumberland Ankle Instability Tool scale after applying an ankle instability training program for 6 weeks to 70 athletes with chronic ankle instability. Additionally, based on the results of the Star Excursion Balance Test (SEBT), the values pre- and post-intervention showed an increase in the anterior direction from (79.69 ± 1.41 cm) to (79.73 ± 1.39 cm), in the posteromedial direction from (83.54 ± 1.27 cm) to (83.55 ± 1.27 cm), and in the posterolateral direction from (79.89 ± 1.07 cm) to (79.89 ± 1.06 cm). These results were similar to the results of the Y balance test we conducted. Considering these findings, the implementation of the sonic balance pad demonstrated an accelerated effect on dynamic balance and produced similar results within a shorter timeframe of 4 weeks as opposed to 6 weeks.

A randomized controlled trial by Wright et al. (2016) [8] compared the effects of training to stand on one leg on a stability board for 4 weeks with an exercise program using elastic bands in 40 adults with ankle instability. As a result, training on the balance board showed significant improvements in ankle instability, function, quality of life, dynamic balance (SEBT- posteromedial 6.5% improvement), and static balance (the foot lift test 29.3% improvement). In this study, both groups showed significant improvement in proprioception and Y balance tests pre- and post-intervention (*p* < 0.05). This reflects the effect of the ankle stabilization exercise program applied in both groups and is thought to be effective in improving proprioception and Y balance testing in patients with ankle instability.

Guzman et al. (2018) [36] studied 48 athletes with ankle instability to find out how much a 6-week Whole Body Vibration (WBV) training program on an unstable surface had a positive effect on balance and body composition in athletes with CAI. Sonic whole-body vibrator and BOSU were used for exercise three times a week for 6 weeks. After 6 weeks of training, improvements in the Biodex Balance System occurred only on the Overall Stability Index (*p* < 0.05) and Anterior–Posterior Stability Index (*p* < 0.05) in the vibration group. They observed better performance in the medial (4.93 ± 3.78%) and posterolateral (5.21 ± 9.43%) directions and composite score of the SEBT in the vibration group and they also observed better performance in the medial (7.36 ± 10.34%), posteromedial (8.75 ± 13.53%), and posterolateral (5.32 ± 7.93%) directions and composite score of SEBT in the non-vibration (NVIB) group. In the vibration group, significant decreases were observed between pre and post1 in the Overall Stability Index (OSI) by −18.69 ± 21.58% and the Anterior–Posterior Stability (APSI) Index by −13.28 ± 25.34%. Similarly, significant decreases were observed between pre and post2 in the OSI by −20.14 ± 24.62% and the APSI by −15.34 ± 27.42%. However, given that the improvements in the Biodex Balance System test performance were only found in the VIB group, we can conclude that the addition of vibration led to different improvements in balance ability compared to the same intervention without vibration. Furthermore, the results obtained from our research provide additional support for the hypothesis derived from this thesis.

In this study, there was no significant difference between the two groups pre- and post-intervention in all variables of the static standing balance test. Moreover, there was no significant difference in the amount of change between the groups. This is thought to be due to the characteristics of the exercise program. All exercise programs implemented were performed on an unstable support surface. It is also thought to be an intense dynamic training program that involves extending the arms forward or sitting and standing up. As a result, it is thought that there is no significant change in static ability.

There are several limitations to the conduct of this study. First, the average age of the subjects participating in the study was 20 years, so it was difficult to generalize to a wide age range of subjects with ankle instability. This may result in sampling bias (convenience sampling was used), as participants are not randomly selected from a larger population. This may also result in a lack of variety in the sample. Another limitation was that there was a limitation in objectively presenting the expected effect of the exercise, as there was a difference in the number of repetitions between the subjects in the ankle stabilization exercise program according to the difference in muscle strength and endurance. Finally, generalizing the results of the study is challenging due to the similarity in physical characteristics and ages among the subjects used. Therefore, a follow-up study in the future is required to confirm its effects on subjects across different age groups and with diverse physical characteristics.

## 8. Conclusions

In conclusion, both groups showed significant changes in the detailed dependent variables of proprioception, YBT, and long jump test. When the two groups were compared, it was seen that the amount of change in the sonic balance pad was more than in the balance pad. Considering all these results, we concluded that ankle stabilization exercise on unstable ground is effective for ankle stabilization by effectively increasing the proprioceptive sense and dynamic balance ability in patients with ankle instability, and the effect increases when sound waves are applied together. We also concluded that the addition of vibration led to different improvements in balance ability compared to the same intervention without vibration. Furthermore, the results obtained from our research provide additional support for the hypothesis derived from this thesis.

## Figures and Tables

**Table 1:** Training exercise.

Exercise	Timing, Reps	Sets	Rest Interval	Figures
Warm-up	≥10 min			
Rotate the neck in one direction, rotate the arms, rotate the hip joint, and rotate the ankle joint while lifting one leg (Approximately 30 s)				
Brisk Walking	1 min	3 sets	10 s	
Proprioceptive Strengthening Exercise				
Part 1: One leg standing				Figure 2A
After one leg was worked for 30 s and rested for 10 s, the exercise was continued for the opposite leg under the same conditions. The alternating exercise performed on both legs was considered as one set.		4 sets	30 s	
Part 2: One leg heel-up				Figure 2B
The alternating exercise performed on both legs was considered as one set.	15 reps per set	4 sets	30 s	
Strength Training				
Part 1: Squat				Figure 2C
The alternating exercise performed on both legs was considered as one set.	20 reps per set	4 sets	30 s	
Part 2: One leg dead lift				Figure 2D
The alternating exercise performed on both legs was considered as one set.	10 reps per set	4 sets	30 s	
Cool-down	≥3 min			

**Table 2:** General characteristics of all the subjects (*n* = 30).

Characteristics	Balance Pad (*n* = 15)	Sonic Pad (*n* = 15)	X^2^ or *t* (*p*)
Gender (Male/Female)	2/13	7/8	−1.959 (0.050)
Age (years)	20.33 ± 1.18	21.27 ± 1.47	−1.896 (0.058)
Height (cm)	162.80 ± 5.98	164.73 ± 10.91	−0.250 (0.803)
Weight (kg)	57.93 ± 7.65	62.13 ± 13.23	−0.540 (0.589)

**Table 3:** The comparisons of proprioception (*n* = 30).

Items	Groups	Mean ± SD			z (*p*)
		Pre	Post	Post-Pre	
LDE	Balance pad (*n* = 15)	2.27 ± 1.16	1.67 ± 1.29	−0.37 ± 1.32	−2.724 (0.006)
	Sonic pad (*n* = 15)	2.87 ± 1.25	1.67 ± 1.11	−1.43 ± 1.40	−2.539 (0.011)
	*t* (*p*)	−1.222 (0.222)	−0.066 (0.948)	−1.890 (0.059)	
LPE	Balance pad (*n* = 15)	2.00 ± 1.36	2.73 ± 1.22	.60 ± 1.78	−0.785 (0.432)
	Sonic pad (*n* = 15)	3.53 ± 1.25	2.20 ± 1.01	−1.23 ± 1.59	−2.472 (0.013)
	*t* (*p*)	−2.778 (0.005)	−1.466 (0.143)	−2.583 (0.010)	
RDE	Balance pad (*n* = 15)	2.47 ± 0.92	2.33 ± 1.40	0.03 ± 1.32	−3.013 (0.003)
	Sonic pad (*n* = 15)	3.27 ± 1.39	1.20 ± 0.77	−2.13 ± 1.29	−3.161 (0.002)
	*t* (*p*)	−1.612 (0.107)	−2.532 (0.011)	−3.483 (<0.001)	
RPE	Balance pad (*n* = 15)	2.87 ± 1.55	2.07 ± 1.22	−0.63 ± 1.67	−3.259 (0.001)
	Sonic pad (*n* = 15)	3.93 ± 1.83	2.27 ± 1.16	−1.63 ± 1.55	−2.689 (0.070)
	*t* (*p*)	−1.724 (0.085)	−0.370 (0.711)	−1.507 (0.132)	

LDE: Left dorsiflexion error, LPE: Left plantar flexion error, RDE: Right dorsiflexion error, RPE: Right plantar flexion error.

**Table 4:** The comparisons of static standing balance (*n* = 30).

Items	Groups	Mean ± SD			z (*p*)
		Pre	Post	Post-Pre	
Eyes open					
Moving Distance (mm/s)	Balance pad (*n* = 15)	33.36 ± 7.53	31.20 ± 5.48	−2.15 ± 9.88	0.845 (0.412)
	Sonic pad (*n* = 15)	34.09 ± 6.49	35.46 ± 6.07	1.36 ± 6.59	−0.804 (0.435)
	*t* (*p*)	−0.285 (0.778)	−2.017 (0.053)	−1.149 (0.260)	
Moving Speed (mm/s)	Balance pad (*n* = 15)	1.11 ± 0.25	1.04 ± 0.18	−0.07 ± 0.32	0.847 (0.412)
	Sonic pad (*n* = 15)	1.14 ± 0.22	1.18 ± 0.20	0.04 ± 0.21	−0.804 (0.435)
	*t* (*p*)	−0.285 (0.778)	−2.019 (0.053)	−1.148 (0.261)	
Eyes closed					
Moving Distance (mm/s)	Balance pad (*n* = 15)	40.27 ± 10.84	35.45 ± 9.21	−4.82 ± 9.01	2.072 (0.057)
	Sonic pad (*n* = 15)	39.61 ± 8.81	41.25 ± 10.74	1.64 ± 9.38	−0.676 (0.510)
	*t* (*p*)	0.184 (0.855)	−1.587 (0.124)	−1.923 (0.065)	
Moving Speed (mm/s)	Balance pad (*n* = 15)	1.34 ± 0.36	1.18 ± 0.31	−0.16 ± 0.30	2.072 (0.057)
	Sonic pad (*n* = 15)	1.32 ± 0.29	1.37 ± 0.36	0.05 ± 0.31	−0.675 (0.511)
	*t* (*p*)	0.184 (0.855)	−1.587 (0.124)	−1.923 (0.065)	

**Table 5:** The comparisons of Y balance test (*n* = 30).

Items	Groups	Mean ± SD			z (*p*)
		Pre	Post	Post-Pre	
AR (cm)	Balance pad (*n* = 15)	72.30 ± 17.01	71.32 ± 7.97	−0.98 ± 17.54	−2.215 (0.027)
	Sonic pad (*n* = 15)	66.04 ± 6.35	77.86 ± 7.17	11.82 ± 7.41	−3.296 (0.001)
	*t* (*p*)	−1.514 (0.130)	−1.348 (0.178)	−3.402 (0.001)	
AL (cm)	Balance pad (*n* = 15)	72.85 ± 19.95	63.41 ± 8.66	−3.44 ± 19.21	−0.057 (0.955)
	Sonic pad (*n* = 15)	68.16 ± 6.38	76.17 ± 3.67	8.01 ± 6.77	−2.856 (0.004)
	*t* (*p*)	−0.353 (0.724)	−2.592 (0.010)	−2.800 (0.005)	
PMR (cm)	Balance pad (*n* = 15)	78.59 ± 15.34	81.41 ± 10.37	2.82 ± 15.54	−2.045 (0.041)
	Sonic pad (*n* = 15)	86.33 ± 16.44	102.23 ± 11.53	15.96 ± 8.24	−3.296 (0.001)
	*t* (*p*)	−1.224 (0.221)	−4.044 (<0.001)	−3.132 (0.002)	
PML (cm)	Balance pad (*n* = 15)	78.00 ± 16.99	83.36 ± 10.15	5.36 ± 16.78	−2.385 (0.017)
	Sonic pad (*n* = 15)	88.96 ± 19.92	102.45 ± 12.98	13.49 ± 10.91	−3.296 (0.001)
	*t* (*p*)	−2.489 (0.013)	−3.920 (<0.001)	−1.307 (0.191)	
PLR (cm)	Balance pad (*n* = 15)	78.16 ± 14.33	82.61 ± 10.73	4.44 ± 13.15	−2.272 (0.023)
	Sonic pad (*n* = 15)	87.95 ± 17.51	101.34 ± 15.37	13.38 ± 10.79	−3.296 (0.001)
	*t* (*p*)	−1.804 (0.071)	−3.920 (<0.001)	−1.680 (0.093)	
PLL (cm)	Balance pad (*n* = 15)	80.86 ± 14.96	81.31 ± 7.16	0.45 ± 15.40	−0.659 (0.510)
	Sonic pad (*n* = 15)	91.23 ± 17.35	104.18 ± 11.78	12.96 ± 13.32	−3.296 (0.001)
	*t* (*p*)	−2.385 (0.017)	−4.376 (<0.001)	−2.489 (0.013)	

AR: Anterior right, AL: Anterior left, PMR: Posteromedial right, PML: Posteromedial left, PLR: Posterolateral right, PLL: Posterolateral left.

## Data Availability

Data are available upon request to the corresponding author.
